# Investigation of useful carbon tracers for ^13^C-metabolic flux analysis of *Escherichia coli* by considering five experimentally determined flux distributions

**DOI:** 10.1016/j.meteno.2016.06.001

**Published:** 2016-06-07

**Authors:** Kousuke Maeda, Nobuyuki Okahashi, Yoshihiro Toya, Fumio Matsuda, Hiroshi Shimizu

**Affiliations:** Department of Bioinformatic Engineering, Graduate School of Information Science and Technology, Osaka University, 1-5 Yamadaoka, Suita, Osaka 565-0871, Japan

**Keywords:** ^13^C-metabolic flux analysis, Design of experiment, ^13^C-labeling experiment, *Escherichia coli*, Computer simulation

## Abstract

The ^13^C-MFA experiments require an optimal design since the precision or confidence intervals of the estimated flux levels depends on factors such as the composition of ^13^C-labeled carbon sources, as well as the metabolic flux distribution of interest. In this study, useful compositions of ^13^C-labeled glucose for ^13^C-metabolic flux analysis (^13^C-MFA) of *Escherichia coli* are investigated using a computer simulation of the stable isotope labeling experiment. Following the generation of artificial mass spectra datasets of amino acid fragments using five literature-reported flux distributions of *E. coli*, the best fitted flux distribution and the 95% confidence interval were estimated by the ^13^C-MFA procedure. A comparison of the precision scores showed that [1, 2-^13^C]glucose and a mixture of [1-^13^C] and [U-^13^C]glucose at 8:2 are one of the best carbon sources for a precise estimation of flux levels of the pentose phosphate pathway, glycolysis and the TCA cycle. Although the precision scores of the anaplerotic and glyoxylate pathway reactions were affected by both the carbon source and flux distribution, it was also shown that the mixture of non-labeled, [1-^13^C], and [U-^13^C]glucose at 4:1:5 was specifically effective for the flux estimation of the glyoxylate pathway reaction. These findings were confirmed by wet ^13^C-MFA experiments.

## Highlights

• Useful compositions of ^13^C-labeled glucose are investigated for ^13^C-MFA of *E. coli*.
• Computer simulations revealed that [1,2-^13^C] was one of the best first choices.
• Mixture of non-labeled, [1-^13^C] and [U-^13^C] at 0:8:2 was also suitable for ^13^C-MFA.
• Mixture at 4:1:5 was specifically effective for estimation of glyoxylate pathway.
• The wet ^13^C-MFA experiments of *E. coli* confirmed the findings.

## 1 Introduction

The ^13^C-metabolic flux analysis (^13^C-MFA) is a tool for detailed understanding of intracellular carbon flux distributions of microbial and mammalian cells (Antoniewicz, 2013b, Antoniewicz, 2015, Wiechert, 2001, Wittmann, 2007, Zamboni et al., 2009). Following cultivation of the cells in medium containing ^13^C-labeled carbon sources, a metabolic flux distribution was estimated from the ^13^C-labeling patterns of intracellular metabolites (mass isotopomer distribution (MID)) (Antoniewicz et al., 2007b, Zamboni et al., 2009). The ^13^C-MFA has been originally developed for metabolic engineering of microbes (Costenoble et al., 2007, Shirai et al., 2007, Wasylenko and Stephanopoulos, 2015), and recently applied to the quantitative analysis of cell metabolism in various fields of systems biology (Christen and Sauer, 2011, Haverkorn van Rijsewijk et al., 2011, Shimizu, 2004) and cancer research (Gaglio et al., 2011, Hiller and Metallo, 2013).

^13^C-MFA experiments require an optimized experimental design since the precision or confidence interval of the estimated flux levels depends on factors including (i) the structure of the metabolic network model, (ii) the composition of ^13^C-labeled carbon sources, (iii) the available intracellular metabolites for the MID measurement, (iv) the number of experiments performed, and (v) the metabolic flux distribution of interest (Antoniewicz, 2013a). In the case of ^13^C-MFA of *E. coli*, a metabolic model has been well established and a list of available fragment ions has been reported for gas chromatography-mass spectrometry (GC–MS) analysis of amino acids (Antoniewicz et al., 2007a; Okahashi et al., 2014). Despite the best precision for the flux estimation having been attained by introducing parallel labeling experiments using 14 carbon sources (Crown et al., 2015, Leighty and Antoniewicz, 2013), further investigation of the useful ^13^C-labeled carbon sources is still needed since single labeling experiments have been performed for the ^13^C-MFA of *E. coli*.

The selection of ^13^C-labeled carbon tracers has, however, bothered the design of ^13^C-MFA experiments. This is because a strict optimization of ^13^C-labeled carbon sources depends on the metabolic flux distribution inside of the target cells, a factor that is usually unknown before a ^13^C-MFA experiment. Furthermore, the relationship between precision of flux estimations, composition of [^13^C]glucose (tracers) and the metabolic flux distribution remains unclear. Thus, the best first choice of a ^13^C-labeled carbon source for the precise estimation of any given flux distribution in the whole metabolic network is also unclear. Recent optimization efforts via the analysis of experimental data or computer simulation of ^13^C-MFA experiments revealed that [4,5,6-^13^C]glucose and the mixture of [1-^13^C] and [U-^13^C]glucose at a ratio of 8:2 are suitable for the flux determinations of the TCA cycle and the pentose phosphate pathway, respectively (Crown et al., 2015, Millard et al., 2014, Shupletsov et al., 2014). Further investigation considering the other flux distributions is however needed, since the tracer compositions were optimized for one metabolic flux distribution of *E. coli.*

In this study, the relationship among the precision of metabolic flux estimation, composition of [^13^C]glucose and the metabolic flux distribution was investigated using a computer simulation of the ^13^C-labeling experiment of the ^13^C-MFA of *E. coli*. A series of artificial MID datasets of amino acid fragments were generated for various compositions of [^13^C]glucose using the literature reported flux distributions of *E. coli*. Useful compositions of [^13^C]glucose were investigated by comparing 95% confidence intervals of estimated flux distributions. Similar approach was employed to optimize the best composition of ^13^C-glucose for the metabolic flux analysis of *Synechocystis* sp. PCC6803 under the mixotrophic condition (Arauzo-Bravo and Shimizu, 2003). In this study, the simulation was performed for five literature reported flux distributions of *E. coli* to investigate the relationship between the precision of metabolic flux estimation, composition of [^13^C]glucose and the metabolic flux distribution, from which compositions of useful [^13^C]glucose were discussed. The results showed that [1,2-^13^C]glucose and the mixture of [1-^13^C] and [U-^13^C]glucose at 8:2 are the most useful first choices for a precise estimation of flux level, whereas the anaplerotic and glyoxylate pathway reactions are affected by both the carbon source and flux distribution. The simulation results were confirmed experimentally by ^13^C-MFA of *E. coli*.

## 2 Materials and methods

### 2.1 Software

All procedures for the simulation of ^13^C-MFA experiments were performed using a Python version of OpenMebius (Kajihata et al., 2014) implemented in Python 2.7.9 with NumPy 1.9.1, SciPy 0.4.2, PyOpt 1.2, and parallel Python 1.6.4 modules. Metabolic fluxes were estimated by minimizing the residual sum of squares (RSS) between experimentally measured and simulated MIDs using the SLSQP (sequential least squares programming) function implemented in PyOpt 1.2 (Perez et al., 2012). The optimizing function is described as

(1) $$RSS\text{=}\sum_{i\text{=1}}^{N}(\frac{MID_{i}^{measured}-MID_{i}^{estimated}}{\sigma_{i}})$$

where $MID_{i}^{measured}$ is the MID of the *i*th measured metabolite, $MID_{i}^{estimated}$ is the estimated MID of the corresponding metabolite, $\sigma_{i}$ is the standard deviation of MID measurement, and *N* is the number of metabolites used for flux estimation. Multiple jobs for the metabolic flux estimations were executed using the distributed computing function of parallel Python 1.6.4. For all ^13^C-MFA, the flux levels of glucose uptake, excretion of products, and biomass synthesis were fixed to the measured or literature reported values. Ranges of 95% confidence interval were determined by the grid search method (Antoniewicz et al., 2006).

### 2.2 Precision score

Precision scoring was used to evaluate estimation accuracy (Metallo et al., 2009). A normalized range is calculated for each flux using the formula:

(2) $$r_{i}=\min\left(\frac{u_{i}}{\left|v_{i}\right|},\frac{v_{i}}{\left|v_{i}\right|}+1\right)-\max\left(\frac{l_{i}}{\left|v_{i}\right|},\frac{v_{i}}{\left|v_{i}\right|}-1\right)$$

where *u*_*i*_, *v*_*i*_, *l*_*i*_ and *r*_*i*_ are upper bound, the estimated flux, lower bound and normalized range for the *i*th flux. The individual ranges are converted into scores using a negative exponential function.

(3) $$S_{i}=\exp\left(-\frac{r_{i}}{3}\right)$$

The individual score (*S*_*i*_) were summed into an overall score (*S*_*Sum*_).

(4) $$S_{\mathrm{sum}}=\sum_{i}S_{i}$$

Larger *S*_*i*_ and *S*_*Sum*_ levels indicate narrower confidence intervals or more precise estimation of determined flux levels of a reaction and the whole metabolic network, respectively.

### 2.3 Optimization of the ^13^C tracer by computational simulation of ^13^C-MFA

**Fig. 1:**
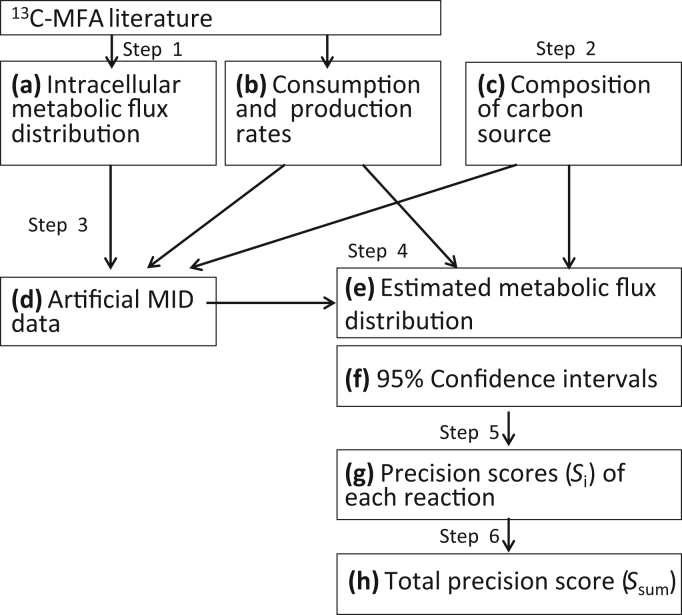
Procedure for computational simulation of ^13^C-MFA experiments. Step 1: The intracellular flux distribution (a) and the consumption and production rate (b) data were obtained from the literature. Step 2: The composition of carbon source was arbitrarily selected (c). Step 3: The theoretical MIDs were calculated and Gaussian noise at 1% levels was added to produce artificially measured MID data (d). Step 4: The metabolic flux distribution (e) and those 95% confidence intervals (f) were estimated. Step 5: An accuracy score *S*_*i*_ (g) was determined for each reaction *i*. Step 6: The sum of *S*_*i*_ of all reactions, *S*_*sum*_, was calculated (h).

The computer simulation of a ^13^C-MFA experiment was conducted by the following procedure (Fig. 1). Step 1: The intracellular flux distribution (Fig. 1a), the consumption and production rates (Fig. 1b) data were obtained from the literature. In this study, the ^13^C-MFA studies for (A) a continuous culture of *E. coli* MG1655 (Okahashi et al., 2014), (B) a batch cultivation of MG1655 (Crown et al., 2015), (C) a batch cultivation of a *pgi*Δ deletion strain (Toya et al., 2010), (D) a batch cultivation of a *pyk*Δ deletion strain (Toya et al., 2010), and (E) a batch cultivation of *E. coli* BW25113 (Toya et al., 2007) were used (Supplementary data S1). Step 2: A composition of [^13^C]glucose was arbitrarily selected (Fig. 1c). Step 3: The theoretical MIDs were calculated for the 24 fragments of amino acids shown in Table 1, and then the Gaussian noise at 1% levels was added to produce artificially measured MID data (Fig. 1d). Step 4: A metabolic flux distribution (Fig. 1e) and its 95% confidence intervals (Fig. 1f) were estimated by the method mentioned above. The standard deviations of MID measurements were set at 0.01. The metabolic networks of *E. coli* consisting of glycolysis, pentose phosphate pathway, TCA cycle, anaplerotic pathway, glyoxylate pathway, and Entner-Doudoroff pathway were employed following the original literature (Supplementary data S1). The metabolic models of the literature data (D) and (E) do not include glyoxylate and Entner-Doudoroff pathway related reactions (Toya et al., 2007, Toya et al., 2010). Step 5: An accuracy score *S*_*i*_ (Fig. 1g) was determined for each reaction *i* by Eq. (3). Step 6: The sum of *S*_*i*_ of all reactions, *S*_*sum*_, was calculated by Eq. (4). The calculation of one *S*_*sum*_ value requires approximately 40 min with this procedure on a PC cluster running Windows Server 2008 (Xeon® CPU E7-8870, L5460 and AMD Opteron™ Processor 6238, 100 cores in total).

**Table 1:** Amino acids fragments used in the simulation of ^13^C-metabolic flux analysis.

	Ala	Val	Leu	Ile	Gly	Ser	Phe	Tyr	Asp	Thr	Glu
[M-57]^+^	**+**	**+**			**+**	**+**	**+**		**+**	**+**	**+**
[M-85]^+^	**+**	**+**	**+**	**+**	**+**	**+**	**+**		**+**		
[M-159]^+^				**+**		**+**	**+**		**+**		**+**
[f302]^+^							**+**	**+**	**+**		

### 2.4 ^13^C-metabolic flux analysis

[1-^13^C]glucose (99%) and [U-^13^C]glucose (99%) were purchased from Cambridge Isotope Laboratory (Andover, MA). *E. coli* K-12 MG1655 was precultured using 100 mL of M9 medium containing 3 g/L glucose in 500 mL Sakaguchi flasks at 37 °C with shaking at 120 rpm. At OD_600_=1.0, the preculture was transferred to the main cultures. The main cultures were carried out at identical conditions (initial OD_600_=0.01), except for using mixtures of non-labeled, [1-^13^C], and [U-^13^C]glucose at a ratio of 0:8:2 and 4:1:5 as the carbon sources. Biomass samples were taken 6.5 h after main cultures started. The concentrations of cells, glucose, and acetate in the culture medium were analyzed by the previously described method (Okahashi et al., 2014).

The analysis of ^13^C-labeled proteinogenic amino acids was performed as described previously (Antoniewicz et al., 2007a, Okahashi et al., 2014). In short, proteinogenic amino acids were obtained by acid hydrolysis of cells in 10 mL of culture broth and derivatized by *N-*(*tert*-butyldimethylsilyl)-*N*-methyl-trifluoroacetamide containing 1% *tert*-butyldimethylchlorosilane. The MIDs of ion clusters of 24 fragments in Table 1 were determined by GC–MS (Agilent 7890 GC and Agilent 5975 MSD (Agilent Technologies). The MID data of [M-85]^+^ of leucine (Leu), [M-57] ^+^ and [M-85] ^+^ of glycine (Gly), [M-85]^+^ of serine (Ser), and [M-57] ^+^ and [M-157] ^+^ of glutamine (Gln) were not employed due to large standard deviations in the mass analysis. The effect of naturally occurring isotopes was removed from the raw mass spectrometry data to obtain corrected ^13^C-labeling patterns of the carbons in the amino acids (van Winden et al., 2002). The fluxes for biomass synthesis of *E. coli* were calculated from the precursor requirement. The standard deviations of MID measurements were set at 0.007. The metabolic flux distribution was estimated using the metabolic networks of *E. coli* consisting of glycolysis, pentose phosphate pathway, TCA cycle, anaplerotic pathway, glyoxylate pathway, and Entner-Doudoroff pathway (Supplementary data S4).

## 3 Results

### 3.1 Computer simulation of ^13^C-MFA of *E. coli* using [1-^13^C] and [U-^13^C]glucose

Various mixtures of non-labeled, [1-^13^C], and [U-^13^C]glucose such as 0:8:2, 0:5:5, and 5:3:2 have been widely employed in previous ^13^C-MFA studies of *E. coli* due to the commercial availability of [1-^13^C] and [U-^13^C]glucose (Crown and Antoniewicz, 2013). In this study, a computer simulation of ^13^C-MFA was conducted to investigate the relationship among the precision of metabolic flux estimation, compositions of [^13^C]glucose and the metabolic flux distribution (Fig. 1). This study considers the ^13^C-MFA of the central carbon metabolism in *E. coli* cultured in a medium containing glucose as sole carbon source, where the metabolic flux distribution at an isotopically steady state is estimated by a single labeling experiment using the MID data of the proteinous amino acids.

**Fig. 2:**
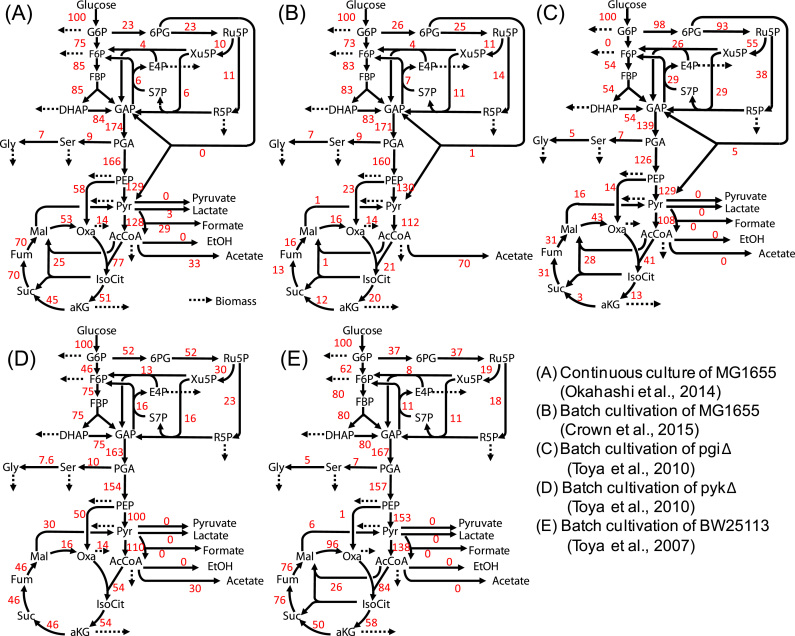
Experimentally determined metabolic flux distributions used in this study. Flux values (red numbers) are normalized to a glucose uptake rate of 100. (For interpretation of the references to color in this figure legend, the reader is referred to the web version of this article.)

As mentioned in Section 1, the precision of estimated flux levels depends on the structure of the metabolic network model, composition of ^13^C-labeled glucose, available fragments of amino acids for the MID measurement, numbers of experiments, and the metabolic flux distribution of interest. Here, artificial MID data of 24 fragment ions of the amino acids in Table 1 were used throughout the study. These fragment ions are commonly employed among previous ^13^C-MFA studies (Antoniewicz et al., 2007a, Crown et al., 2015, Okahashi et al., 2014, Toya et al., 2010). The useful carbon sources were investigated from all patterns of non-labeled, [1-^13^C], and [U-^13^C]glucose mixtures with 10% step size (66 patterns in total). Furthermore, simulations of single labeling experiments were performed for five distinct metabolic flux distributions of *E. coli* (Fig. 2) determined from (A) a continuous culture of MG1655 (Okahashi et al., 2014), (B) a batch cultivation of MG1655 (Crown et al., 2015), (C) a batch cultivation of the *pgi*Δ deletion strain, (D) a batch cultivation of the *pyk*Δ deletion strain (Toya et al., 2010), and (E) a batch cultivation of *E. coli* BW25113 (Toya et al., 2007). Metabolic models were also obtained from the literature (Fig. 2 and Supplementary data S1).

**Fig. 3:**
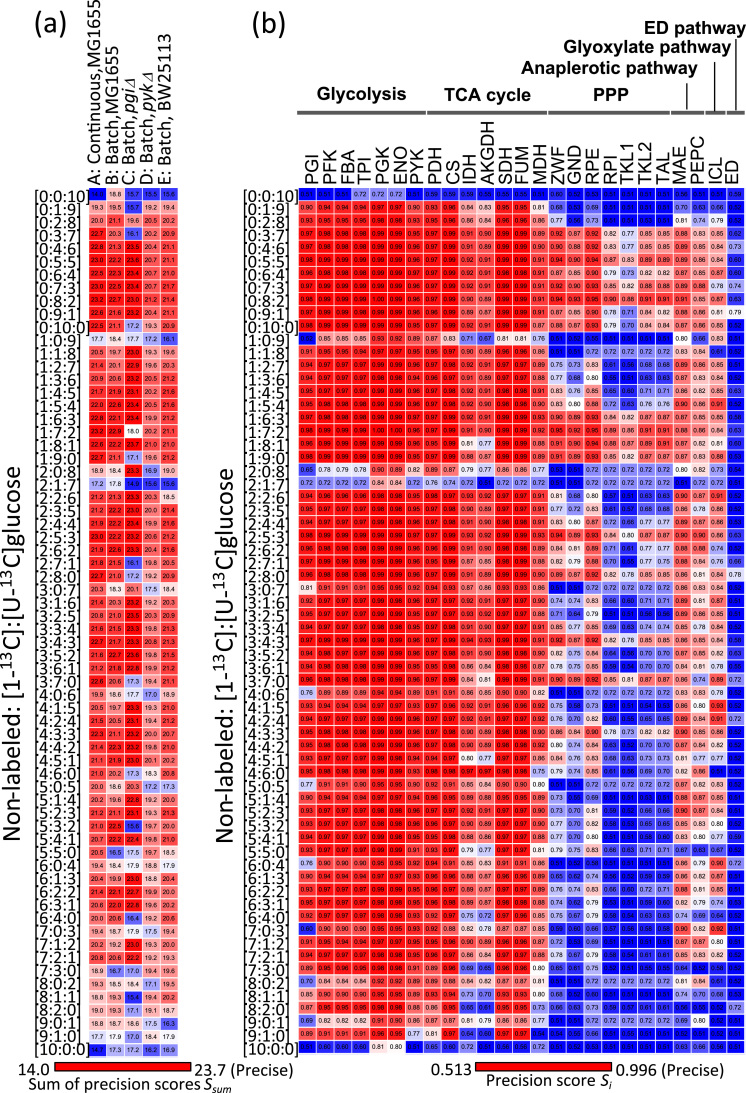
Heatmap representations of the precision scores *S*_*i*_ and *S*_*sum*_ levels estimated by the computer simulation of ^13^C-MFA using 66 mixtures of non-labeled, [1-^13^C], and [U-^13^C]glucose as carbon sources. (a) Comparison of *S*_*sum*_ levels determined for the five metabolic flux distributions. (b) Precision scores *S*_*i*_ of each reaction determined for the metabolic flux distribution A (a continuous culture of *E. coli* MG1655). The red and blue colors in the boxes represent larger (better precision) and smaller (poorer precision) *S*_*sum*_ levels of flux estimation, respectively. (For interpretation of the references to color in this figure legend, the reader is referred to the web version of this article.)

For example, the intracellular flux distribution, the glucose consumption, and product excretion rates of MG1655 under the continuous culture condition was obtained from the literature (Okahashi et al., 2014) (step 1 in Fig. 1). The composition of non-labeled, [1-^13^C], and [U-^13^C]glucose in the carbon source was arbitrarily set to 0:8:2 (step 2 in Fig. 1). A theoretical MID of [M-57]^+^ fragment for alanine (*m*/*z* 260–263) was calculated to be [0.38, 0.36, 0.12, 0.15] from the experimentally determined flux distribution (Fig. 1a), specific rates, (Fig. 1b), and the composition of carbon sources (Fig. 1c) (step 3). The theoretical MIDs were calculated for 24 fragments of the amino acids in Table 1, to which the Gaussian noise at 1% levels was added to produce artificially measured MID data (Fig. 1d). The metabolic flux distribution (Fig. 1e) and its 95% confidence intervals (Fig. 1f) were estimated by the procedure of ^13^C-MFA using artificial MID data (Fig. 1d) (step 4). Furthermore, an accuracy score *S*_*i*_ (Fig. 1g) was determined for each reaction *i*, where a larger *S*_*i*_ score indicates narrower 95% confidence interval or more precise estimation of flux level (see Material and Methods for detailed definition, step 5 in Fig. 1) (Metallo et al., 2009). In the case of the flux distribution of (A) with a glucose ratio at 0:8:2, the metabolic flux level and the 95% confidence interval of the reaction glucose-6-phosphate (G6P) -> 6-phosphogluconate (6PG) were estimated to be 24.6 and 23.5–27.7, respectively, from which *S*_*G6P ->6PG*_ was determined to be 0.94. Finally, the sum of *S*_*i*_ of all reactions *S*_*sum*_ was determined to be 23.24 (Fig. 1h). Here, the *S*_*sum*_ values were determined by this procedure for all 330 combinations of the mixtures of carbon sources and the five flux distributions (Fig. 3). In this study, the precision scores were determined from one replicate of the simulation since deviations of *S*_*sum*_ level (mean±standard deviation was estimated as 23.05±0.39 from 10 iterative simulations of the above procedure, for example) were smaller than the range of observed *S*_*sum*_ levels (approximately 14–24, Fig. 3).

Fig. 3a shows a heatmap representation of the *S*_*sum*_ levels determined for the five metabolic flux distributions. The red and blue colors represent larger (better precision) and smaller (poorer precision) *S*_*sum*_ scores of overall flux estimation, respectively. This result indicates that there are variations in *S*_*sum*_ levels depending on the carbon source mixtures and the metabolic flux distributions. For example, mixture ratios such as at 0:10:0 (100% [1-^13^C]glucose) and 5:0:5 (50% [U-^13^C]glucose) of non-labeled, [1-^13^C], and [U-^13^C]glucose showed smaller *S*_*sum*_ values for the flux distributions (C) and (D), respectively. On the other hand, the largest *S*_*sum*_ values were commonly observed for mixture ratios such as at 0:8:2 and 0:5:5. These results indicate that the mixture of 0:8:2 is suitable for a flux estimation of the whole metabolic network, at least for flux distributions similar to those examined in this study.

The precision scores of each reaction (*S*_*i*_) deduced by the computer simulation of the ^13^C-MFA of the flux distribution (A) are shown in Fig. 3b. The comparison of the *S*_*i*_ levels indicates that the metabolic flux levels were more precisely determined for the reactions in glycolysis and the TCA cycle by many [^13^C]glucose mixtures. In contrast, the precision scores vary among the [^13^C]glucose mixtures for the cases of pentose phosphate pathway (PPP), anaplerotic, and the glyoxylate pathway reactions. For instance, the mixture ratios at 5:0:5 and 0:10:0 were less favorable than 0:8:2 and 0:5:5 due to poor precisions in the flux estimation of PPP and anaplerotic reactions. Furthermore, the mixture ratio at 4:1:5 is useful for the flux estimation of the isocitrate lyase (ICL) reaction in the glyoxylate pathway, since the precision score determined by the mixture ratio at 4:1:5 (*S*_*ICL*_=0.93) was larger than that determined by the mixture ratio at 0:8:2 (*S*_*ICL*_=0.87) (Fig. 3b). Similar trends were commonly observed among other flux distributions (Supplementary data S2 and 3). These results suggest that the selection of suitable carbon sources is important for the precise estimation of metabolic flux levels of PPP, anaplerotic, and glyoxylate pathway reactions in the ^13^C-MFA of *E. coli* using a mixture of non-labeled, [1-^13^C] and [U-^13^C]glucose.

### 3.2 Evaluation of all possible ^13^C-labeled types of glucose as carbon sources

**Fig. 4:**
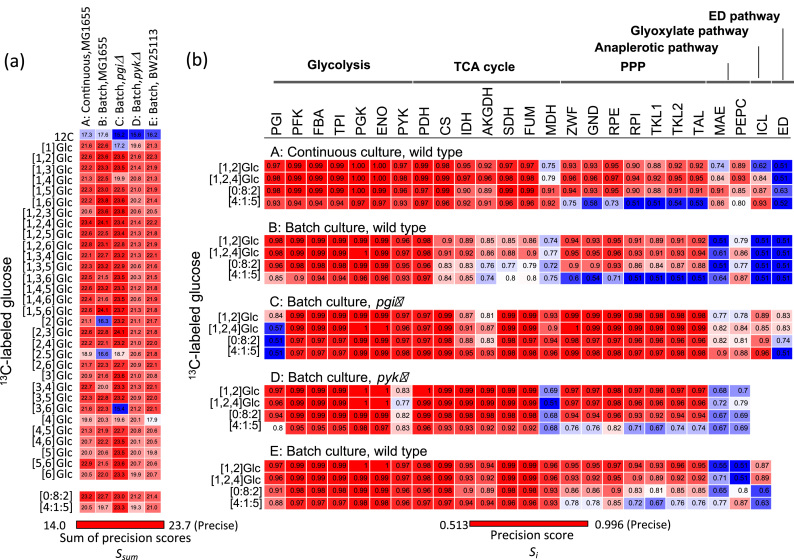
Heatmap representations of the precision scores *S*_*i*_ and *S*_*sum*_ levels estimated by the computer simulation of ^13^C-MFA using all the possible patterns of ^13^C-labeled glucose as carbon source. All results are shown in Supplementary data S2 and S3. (a) Comparison of *S*_*sum*_ levels determined for the five metabolic flux distributions. (b) Precision scores *S*_*i*_ of each reactions determined for the five metabolic flux distributions using [1,2-^13^C] and [1,2,4-^13^C]glucose. The red and blue colors represent larger (better precision) and smaller (poorer precision) *S*_*sum*_ levels of flux estimation, respectively. The precision scores of non-labeled, [1-^13^C], and [U-^13^C]glucose at ratios of 0:8:2 and 4:1:5 are also shown for comparison. (For interpretation of the references to color in this figure legend, the reader is referred to the web version of this article.)

A useful carbon source for the flux estimation of the PPP and the anaplerotic reactions was investigated by additional simulations of ^13^C-MFA to test all possible ^13^C-labeling patterns of glucose as a sole carbon source (Fig. 4 and Supplementary data S2 and S3). The simulation result showed that the largest sum of precision scores *S*_*sum*_ was observed when using [1, 2, 4-^13^C] and [1, 2-^13^C]glucose as carbon sources (Fig. 4a). The *S*_*sum*_ scores were similar or larger than those of the mixture ratio of non-labeled, [1-^13^C], and [U-^13^C]glucose at 0:8:2. In addition to the glycolysis and TCA cycle reactions, the ^13^C-MFA using [1, 2, 4-^13^C] and [1, 2-^13^C]glucose could precisely estimate the flux levels of PPP reactions for five flux distributions (Fig. 4b). The precision should be derived from the fact that three distinct isotopomers ([1, 2-^13^C], [1, 3-^13^C], and [3-^13^C]) of fructose-6-phosphate could be produced from [1, 2-^13^C]glucose through the Embden–Meyerhof-Parnas and two PP pathways, respectively (Supplementary Fig. S1). A previous computational analysis reported that [1, 2^-^^13^C]glucose was a better carbon source than the mixture ratio of non-labeled, [1-^13^C], and [U-^13^C]glucose at 0:8:2 (Schellenberger et al., 2012). This is probably because a Monte Carlo sampling of theoretically feasible flux distributions without evaluating the confidence intervals was employed for finding an optimal carbon source. Our results suggested that [1, 2, 4-^13^C] and [1, 2-^13^C]glucose are useful carbon sources for the precise flux estimation of the PPP. On the other hand, there are large variations in the precision scores of the flux estimation of the anaplerotic and glyoxylate pathway reactions, suggesting that the composition of carbon source affects the precisions scores by significantly interacting with the intracellular flux distributions.

### 3.3 Experimental confirmation by ^13^C-MFA of *E. coli*

**Fig. 5:**
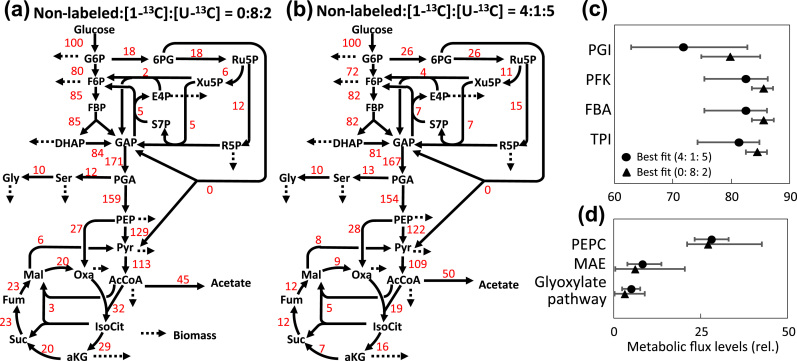
^13^C-metabolic flux analysis of batch cultivated *E. coli* MG1655. (a and b) The metabolic flux distributions estimated from the labeling experiment using mixtures of non-labeled, [1-^13^C], and [U-^13^C]glucose at 0:8:2 (a) and 4:1:5 (b), respectively. (c and d) The 95% confidence intervals of estimated flux levels of the reactions in glycolysis, the anaplerotic and the glyoxylate pathways. Flux values are normalized to a glucose uptake rate of 100.

The simulation of ^13^C-MFA showed that the mixture of non-labeled, [1-^13^C], and [U-^13^C]glucose at 0:8:2 is one of the best first choices for the precise flux analysis of the whole metabolic network of *E. coli*. It was also shown that the mixture ratio at 4:1:5 enabled a precise flux estimation specifically for the glyoxylate pathway. The findings were confirmed by wet ^13^C-MFA experiments. The wild type (MG1655) strain of *E. coli* was batch cultivated under aerobic conditions in M9 media containing non-labeled, [1-^13^C], and [U-^13^C]glucose at 0:8:2. The specific rates for cell growth, glucose consumption, and acetic acid production were determined to be 0.73±0.00 h^−1^, 8.91±0.53, and 4.05±0.41 mmol g-dry cell weight^−1^ h^−1^ from the time-course analysis of the medium composition (Supplementary data S5). No production of ethanol and other organic acids was observed. Following the sampling of *E. coli* cells at an exponential growth phase (OD_600_ ~1.0), MIDs of proteinous amino acids were determined by using the GC–MS (Supplementary data S4). Fig. 5A shows the metabolic flux distribution estimated from the MID and the material balance data. The residual sum of square (RSS) was 58.8, passing the χ^2^ test (Supplementary data S6).

The ^13^C-metabolic flux analysis was also performed by using medium containing non-labeled, [1-^13^C], and [U-^13^C]glucose at 4:1:5. Although essentially identical conditions were employed for this experiment, the estimated distribution was different from the above results in terms of the TCA cycle and PPP reactions (Fig. 5b, Supplementary data S4 and S6, RSS is 21.5 with a *p*-value of 0.999 by χ^2^ test). It was probably due to a slight difference in the aeration conditions between the two distinct experiments since the specific rates for cell growth, glucose consumption, and acetic acid production were 0.70±0.02 h^−1^, 8.13±0.76, and 4.14±0.18 mmol g-dry cell weight^−1^ h^−1^, respectively (Supplementary data S5).

The 95% confidence intervals of all reactions were determined by the grid search method (Fig. 5c, d, and Supplementary Fig. S2). The comparison showed that the ^13^C-MFA using the mixture ratio of 0:8:2 could more precisely estimate the metabolic flux levels of the reactions in the glycolysis since the 95% confidence intervals were narrower than those estimated by the mixture ratio at 4:1:5. For example, the 95% confidential intervals of phosphofructokinase (PFK) were determined to be 83.5–87.1 and 75.4–86.2 by using the mixtures of non-labeled, [1-^13^C], and [U-^13^C]glucose at 0:8:2 and 4:1:5, respectively (Fig. 5c). Similar results were observed for the PPP and TCA cycle reactions (Supplementary Fig. S2). On the other hand, narrower 95% confidence intervals of reactions related to the anaplerotic and glyoxylate pathways were observed for the mixture ratio at 4:1:5. For example, the 95% confidence interval of glyoxylate pathway flux (2.2–7.7) determined by the 4:1:5 mixture was narrower than that determined by the 0:8:2 mixture (0.1–8.8) (Fig. 5d). These results experimentally support the results of the computer simulation of ^13^C-MFA.

## 4 Discussion

The computer simulation of ^13^C-MFA experiments of *E. coli* was performed to investigate the relationship between the precision of metabolic flux estimation, composition of [^13^C]glucose and the metabolic flux distribution. The simulation results showed that the selection of carbon source mainly affected the precision of metabolic flux estimation of PPP, anaplerotic, and glyoxylate pathway reactions. This result also supports that, as has been proposed in previous studies, [1, 2-^13^C]glucose, and the mixture of non-labeled, [1-^13^C], and [U-^13^C]glucose at 0:8:2 are the most suitable carbon tracers for a precise estimation of flux levels of the PPP in addition to glycolysis and the TCA cycle (Crown et al., 2015, Shupletsov et al., 2014). The mixture of non-labeled, [1-^13^C], and [U-^13^C]glucose at 0:8:2 has been used for the ^13^C-MFA of *E. coli* (Fischer et al., 2004). The mixture is an affordable carbon source for the routine metabolic flux analysis due to the relatively lower cost of [1-^13^C]glucose (~$100/g) and [U-^13^C]glucose (~$200/g). However, it has been reported that the isotopic discrimination by microorganisms and isotopic impurities in^13^C-glucose could affect the result of ^13^C-MFA since *E. coli* metabolism selectively incorporates and utilizes light isotopes (Feng and Tang, 2011). The ^13^C-MFA using single ^13^C-glucose has an advantage in this regard, but requires more expensive carbon sources such as [1,2-^13^C]glucose ($500–$1000/g). [1, 2-^13^C]glucose has been employed for the precise metabolic flux analysis of *E. coli* (He et al., 2014), yeast (Wasylenko and Stephanopoulos, 2015), and the flux analysis of the PPP in plants (Nargund and Sriram, 2013). Although the generality has not been guaranteed, these carbon sources could be the best first choice for the ^13^C-MFA of *E. coli* since the results were confirmed by the simulation using five distinct metabolic flux distributions (Fig. 3, Fig. 4) and the wet ^13^C-MFA experiment of the batch cultivated *E. coli* (Fig. 5). The ^13^C-glucose should also be available to other microorganisms with similar metabolic pathways such as *Bacillus subtilis* and *Corynebacterium glutamicum*.

The results also revealed that there are large variations in the precisions scores of the anaplerotic and glyoxylate reactions among the flux distributions. It was predicted from the simulations that the mixture of non-labeled, [1-^13^C], and [U-^13^C]glucose at 4:1:5 was particularly effective for the flux estimation of the glyoxylate pathway reaction (Fig. 3b). Although the finding was confirmed by the wet experiment (Fig. 5), the carbon source could not be versatile enough for any flux distribution since the precision scores of the anaplerotic and glyoxylate pathway reactions are sensitively by both the carbon source and the flux distribution (Figs. 3b and 4b).

In addition to the ^13^C-MFA using the proteinogenic amino acids, the direct analysis of glycolytic intermediates by liquid chromatography (LC)-MS has been attempted to analyze mammalian cell cultures and non-growing microbial cells (Ahn and Antoniewicz, 2013, Metallo et al., 2009, Walther et al., 2012). The best ^13^C-labeled carbon source should be selected for each ^13^C-MFA experiment using glycolytic intermediates, with considering a list of the measurable intermediates under the physiological conditions of targeted cells. However, it is expected that [1, 2-^13^C]glucose or a mixture of [1-^13^C] and [U-^13^C]glucose at 8:2 are also useful carbon tracers. It is because the LC-MS analysis usually produces ^13^C-labeling data equivalent to the precursors of amino acids listed in Table 1 (pyruvate, 3-phosphoglycerate, phopsho*enol*pyruvate, erythrose-4-phosphate, oxaloacetate, and 2-ketoglutarate). Indeed, [1, 2-^13^C]glucose has been used for the metabolic flux analysis of cultured mammalian cells as an optimized ^13^C-labeled carbon source (Ahn and Antoniewicz, 2013, Metallo et al., 2009, Walther et al., 2012).

## 5 Conclusions

The present study suggests that ^13^C-MFA experiments should be designed considering an interaction among precision of flux estimation, carbon sources, and intracellular flux distributions. Following the ^13^C-MFA experiment using the first choice carbon tracers such as [1, 2-^13^C]glucose or a mixture of [1-^13^C] and [U-^13^C]glucose at 8:2, the best carbon tracer for the estimated metabolic flux distribution should be investigated by employing optimization methods such as EMU basis vectors (Crown and Antoniewicz, 2012) and Isodesign (Millard et al., 2014), when more precision is needed in the flux estimation of the anaplerotic and the glyoxylate pathways. Precise flux estimation would be attained by the second ^13^C-MFA employing an optimized single or parallel labeling experiments.

## Footnotes

Appendix A Supplementary data associated with this article can be found in the online version at 10.1016/j.meteno.2016.06.001.
