# Supplementary material for: Investigation of useful carbon tracers for 13C-metabolic flux analysis of Escherichia coli by considering five experimentally determined flux distributions
Source: Metab Eng Commun. 2016 Jun 7;3:187–95. doi: 10.1016/j.meteno.2016.06.001 (PMC5678827; doi:10.1016/j.meteno.2016.06.001)
Supplement: Supplementary file 2 — Supplementary material [file mmc2.doc]

**Supplementary Fig. S1.** ^13^C-labeling of fructose-6-phosphate (F6P) using [1,2-^13^C]glucose as carbon source. Closed and open circles represent ^13^C- and ^12^C-glucose atoms, respectively. Three isotopomers ([1,2-^13^C], [1,3-^13^C], and [1-^13^C]) could be produced at the C_1_-C_2_-C_3_ moieties of F6P via different pathways. The labeling scheme ignores the reverse reactions and other isotopomers of glyceraldehyde-3-phosphate (GAP) for simplification.


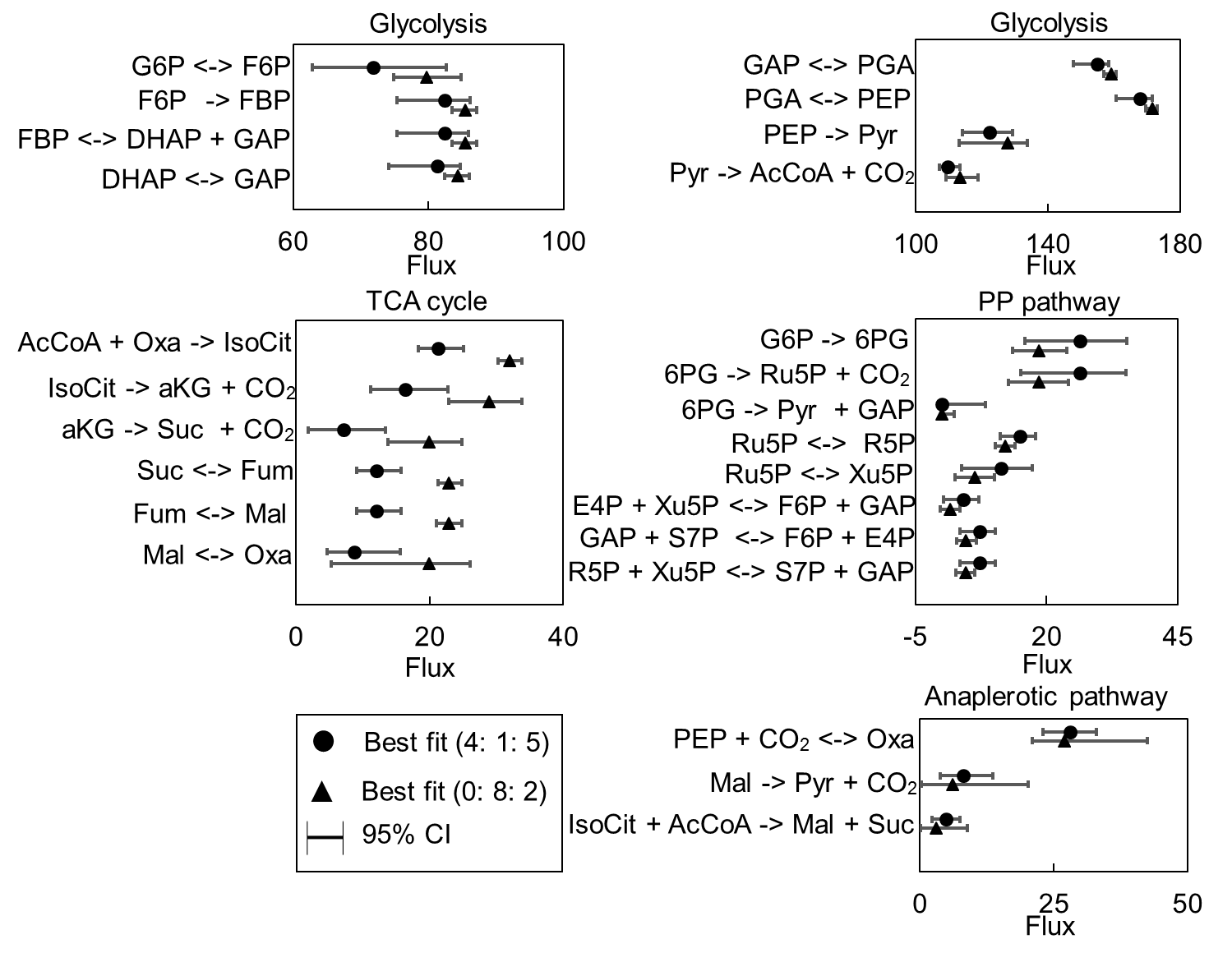


**Supplementary Fig. S2**. 95% confidence intervals of flux levels estimated by the ^13^C-metabolic flux analysis of batch cultivated *E. coli* MG1655 in the labeling experiment using mixtures of non-labeled, [1-^13^C], and [U-^13^C]glucose at 0:8:2 and 4:1:5. The results of glycolysis, TCA cycle, PP Pathway, anaplerotic and glyoxylate pathways are represented. Flux values are normalized to a glucose uptake rate of 100.
